# Etiology and risk factors for diarrheal disease amongst rural and peri-urban populations in Cambodia, 2012–2018

**DOI:** 10.1371/journal.pone.0283871

**Published:** 2023-03-31

**Authors:** Gerard C. Kelly, Agus Rachmat, Robert D. Hontz, Marvin J. Sklar, Long Khanh Tran, Chonthida Supaprom, Malen Luy, Sin Lina, Michael J. Gregory, Heng Sopheab, John S. Brooks, Ian W. Sutherland, Karen S. Corson, Andrew G. Letizia

**Affiliations:** 1 Vysnova Partners, Inc., Landover, Maryland, United States of America; 2 AC Investment Co, Contractor for NAMRU-2 Phnom Penh, Phnom Penh, Cambodia; 3 U.S. Naval Medical Research Unit TWO, Singapore, Singapore; 4 U.S. Naval Medical Research Unit TWO, Phnom Penh, Cambodia; 5 National Institute of Public Health, Ministry of Health, Cambodia; Freelance Consultant, Myanmar, MYANMAR

## Abstract

Diarrheal diseases are a leading cause of mortality and morbidity, disproportionally affecting persons residing in low and middle-income countries. Accessing high-resolution surveillance data to understand community-level etiology and risk remains challenging, particularly in remote and resource limited populations. A multi-year prospective cohort study was conducted in two rural and two peri-urban villages in Cambodia from 2012 to 2018 to describe the epidemiology and etiology of acute diarrheal diseases within the population. Suspected diarrheal episodes among participants were self-reported or detected via routine weekly household visits. Fresh stool and fecal swabs were tested, and acute-illness and follow-up participant questionnaires collected. Of 5027 enrolled participants, 1450 (28.8%) reported at least one diarrheal incident. A total of 4266 individual diarrhea case events were recorded. Diarrhea incidence rate was calculated to be 281.5 persons per 1000 population per year, with an event rate of 664.3 individual diarrhea events occurring per 1000 population per year. Pathogenic *Escherichia coli*, *Aeromonas spp*., and *Plesiomonas shigelloides* were the most prevalent bacterial infections identified. Hookworm and *Strongyloides stercoralis* were the predominant helminth species, while *Blastocystis hominis* and *Giardia lamblia* were the predominant protozoan species found. Norovirus genotype 2 was the predominant virus identified. Mixed infections of two or more pathogens were detected in 36.2% of positive cases. Risk analyses identified unemployed status increased diarrhea risk by 63% (HR = 1.63 [95% CI 1.46, 1.83]). Individuals without access to protected water sources or sanitation facilities were 59% (HR = 1.59 [95% CI 1.49, 1.69]) and 19% (HR = 1.19 [95% CI 1.12, 1.28]) greater risk of contracting diarrhea, respectively. Patient-level surveillance data captured in this long-term study has generated a unique spatiotemporal profile of diarrheal disease in Cambodia. Understanding etiologies, together with associated epidemiological and community-level risk, provides valuable public health insight to support effective planning and delivery of appropriate local population-targeted interventions.

## Introduction

Diarrheal diseases are a leading cause of global mortality and morbidity [1, 2], with a disproportionate impact suffered by persons residing in low and middle-income countries (LMIC), where access to health care, safe drinking water, and sanitation are limited [3–6]. Diarrheal diseases remain among the top five causes of mortality in Southeast Asia, accounting for 6% of all deaths [1], despite a decline in the overall disease burden within the last decade. Determining diarrheal diseases etiology is complex, given the large number of potential causative entero-pathogens, high occurrences of mixed infections, challenges associated with various detection methods, and the sensitivity of available diagnostics [7, 8]. Environmental, economic, and behavioral risk factors associated with diarrheal diseases also vary, both geographically and within populations [9–11]. Characterizing the epidemiology and etiology of diarrheal diseases will aid health professionals and policy makers to review existing strategies, guide future health planning, and target appropriate interventions [7, 10–13].

Despite widespread global occurrence of diarrheal diseases, the availability of detailed data to evaluate etiology and risk is often limited, particularly at the community level [5, 13, 14]. Lack of high-resolution epidemiologic data is amplified in areas of highest burden such as remote and resource-poor settings and populations with limited access to healthcare and risk factors could be different when compared to other settings. Patient-level surveillance datasets provide an optimal source to characterize disease incidence, etiology, and associated risk [14].

In Cambodia, the burden of diarrheal disease is significant and remains a leading cause of mortality in children and adolescents and is a top ten contributor to disability-adjusted life years (DALYs) for all ages [1]. To better characterize diarrheal threats in Cambodia, a multi-year prospective cohort study was conducted in four villages. Aims of the study were to monitor and describe the burden of diarrheal diseases among rural and peri-urban populations, and describe the epidemiology and etiology of acute diarrheal infections amongst village cohorts in Kampong Cham and Tbong Khum provinces in Cambodia.

## Methods

### Study site

**Fig 1:**
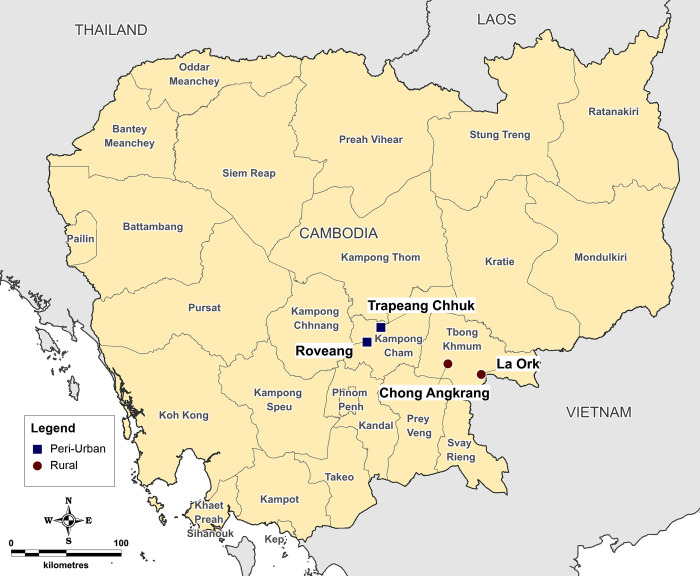
Study site location map. Custom map produced by the authors using MapInfo Professional v15.0.2 (Pitney Bowes Software Inc. 2015, Stamford, CT; https://www.pitneybowes.com) geographic information system mapping software. Site location data were acquired by the study team using global positioning system (GPS). Baseline spatial data were obtained from the GADM database of Global Administrative Areas (https://gadm.org) under a CC-BY 4.0 license [15].

The study was conducted between August 2012 and March 2018, in two peri-urban Cambodian villages in Kampong Cham province (Trapeang Chhuk and Roveang), and two rural villages in Tbong Khumum province (Chong Angkrang and La Ork) (Fig 1). Peri-urban sites in this study were characterized as township-centered settlements with proportionally higher levels of household densities and suburban development, as compared to the rural study site villages characterized by lower household densities and higher proportions of agricultural land. Kampong Cham and Tbong Khumum provinces were selected due to their agricultural-commercial significance within Cambodia and the region, located in the Mekong plains region northeast of the capital Phnom Penh and adjacent to the border with Vietnam. The four representative study site villages within these provinces were selected by the study team due to their geographic diversity, local community interest, and high rates of participation in previous studies.

### Study design

#### Acute disease surveillance program

Participants were recruited for the study through an acute-disease surveillance program established in 2012 as part of a long-term collaboration between the Royal Cambodian MoH, the Cambodian National Institute of Public Health (NIPH), and the United States Naval Medical Research Unit-2 (NAMRU-2). Key objectives of this surveillance program were to support MoH led public health measures through the implementation of high confidence diagnostic testing protocols to measure and monitor rates of febrile vector-borne illness (FVBI), acute respiratory, and diarrheal diseases within the target populations, and identify associated responsible pathogens and risk factors.

#### Participant enrolment and eligibility

Individuals were enrolled into the study during an initial house-to-house census. The study design allowed for the ongoing enrollment of additional participants throughout the course of the observation period as part of annual household information collection and census activities that were conducted in each study village. Inclusion criteria for individuals were age of six months or older, resided in their respective village for six months or more prior to enrolment, physically lived in the village for more than 80% of time throughout the duration of the study, and provided voluntary consent to participate in and comply with all aspects of the study. A meal supplementation, at an approximate value of US$2–3, was offered as an incentive to participants.

#### Data collection and screening

Following enrollment, demographics of each household member, as well as relevant household characteristics pertaining to water, sanitation, hygiene, and the presence of domesticated animals, both pets and livestock, were collected. One adult “primary contact” was identified for each household, was issued a thermometer, and provided instructions on how to identify suspected fever and capture body temperatures of study participants within their household, as well as him or herself, and contacted study staff if a member of the household had diarrhea or other concerning symptoms.

Suspected diarrheal events were reported by the household primary contact to one of 17 local village study field staff who had been trained in interviewing, data gathering, and specimen collection. Routine weekly visits to each household were also conducted by study field staff, where participants were interviewed and asked to self-report any recent occurrences or symptoms of fever or diarrhea. Field staff also tested participants for fever using an axillary thermometer and updated a weekly follow-up log, including household demographics and participant status to maintain an accurate register of study participants upon each study visit.

Individuals confirmed to have had a fever or acute diarrhea, either identified and reported by a primary contact to study field staff, or identified during routine weekly visits, were classified as a case, and visited in their home by a study physician and laboratory assistant for an acute illness investigation. As part of these in-home medical assessments, an acute illness questionnaire was completed and biological specimens including fresh stool and fecal swabs were collected for laboratory testing. Patients who were unable or unwilling to provide a biological specimen during the in-home medical assessment were issued a stool collection kit and asked to provide a specimen within 48 hours. Specific acute illness follow-up visits (approximately 30 and 60 days later) were also conducted. Data collected during these visits included information regarding illness outcomes, additional medical care that may have been sought, such as hospitalization, and any treatments that may have been received.

### Definition of acute diarrhea and fever

For this study, an acute diarrheal case was defined as three or more loose or liquid stools within a 24-hour period, or two loose or liquid stools accompanied by at least two additional gastrointestinal symptoms (such as nausea and/or abdominal cramping) within a 24-hour period, and not lasting more than 14 days. Dysentery was defined as the presence of blood in diarrhea within the previous 10 days. Fever was defined as an axillary temperature ≥37.5°C, or tympanic temperature ≥38°C.

### Specimen collection, transportation, and analysis

Fresh stool and fecal swabs were collected on all study participants. Within one hour of collection, a sample of fresh stool was added to an ova and parasite (O&P) preparation tube for microscopic analysis and molecular characterization. Specimens for microscopic analysis were aliquoted into 10% formalin and polyvinyl alcohol (PVA)-containing medium and transported at room temperature to identify the presence of parasites using microscopy. The remainder of fresh stool was aliquoted into cryovials and transported at 4°C to the NAMRU-2 laboratory, and frozen at -70°C within two hours for future testing by various molecular techniques adapted from previous reports [16–20].

### Data management and statistical analysis

Number of diarrhea cases, proportions, and incidence rates were used to describe the epidemiological and etiological characteristics of the study population. Categorical data were analyzed using Chi-square statistics (expected cell frequency > 5) to measure the association between risk factors and disease and log rank test for survival model to calculate incidence rates (95% confidence interval), with statistical significance set at p < 0.05.

Multi-event survival regression modeling (Cox survival analysis) was used to conduct multi-variate risk analysis and calculate the hazard ratio (HR, 95% confidence interval) and covariate coefficient to the outcome of a diarrhea event [21]. Given the possibility of a participant experiencing diarrhea more than once during the study period, diarrhea cases were considered as recurring events; with an individual diarrhea event treated as a time-varying covariate; and enrolments as multiple failure-time (multiple-event survival) data [22]. To support data modeling, if a participant did not experience a diarrhea event throughout the study, they were assigned a “No” value as a censored status for diarrhea. If a participant had multiple visits without reporting diarrhea, the database dropped all visit observations except for the last visit. If a participant experienced a diarrhea event, they were assigned a “Yes” value for the status of diarrhea. If a participant experienced multiple diarrhea events, then the number of observations for that participant was equal to the number of diarrhea events.

Data were analyzed using Stata software (StataCorp, College Station, TX, USA). Study participants who died or withdrew were right censored upon confirmation.

#### Risk factor variables

To support this data analysis, participant access to water sources were categorized as protected or unprotected. Protected water sources included rainwater, and government regulated water supply wells or tap water. Unprotected water sources included non-regulated sources such as rivers, lakes, or open wells. Sanitation was categorized as access to a toilet facility compared to no access to a toilet facility. Access to a toilet included access to a modern flush toilet, septic, or pit toilet. Treatment of water before drinking was also categorized into treated or untreated, with treated including boiling, filtering, or chemical purification. Untreated water was classified as drinking water directly from its source without intervention. Occupation was categorized as skilled, unskilled, or unemployed, with skilled occupations including business and professional workers, government employees, and students, and unskilled workers including farming, factory, construction, and restaurant workers. Seasonality was categorized as dry and wet seasons, with the wet season typically taking place between May and September, and the dry season from October through April.

### Ethical clearance and consent

The study protocol was approved by the Kingdom of Cambodia’s National Ethics Committee for Health Research and the Naval Medical Research Center’s Institutional Review Board, project number (NAMRU2.2012.0001) in compliance with all applicable federal regulations governing the protection of human subjects. Eligible participants ≥ 18 years of age provided written informed consent, while the parent / legal guardian of non-adult participants (< 18 years of age) provided permission and the dependent assent for participation.

### Inclusivity in global research

Additional information regarding the ethical, cultural, and scientific considerations specific to inclusivity in global research is included in the Supporting Information (S1 Questionnaire)

## Results

**Fig 2:**
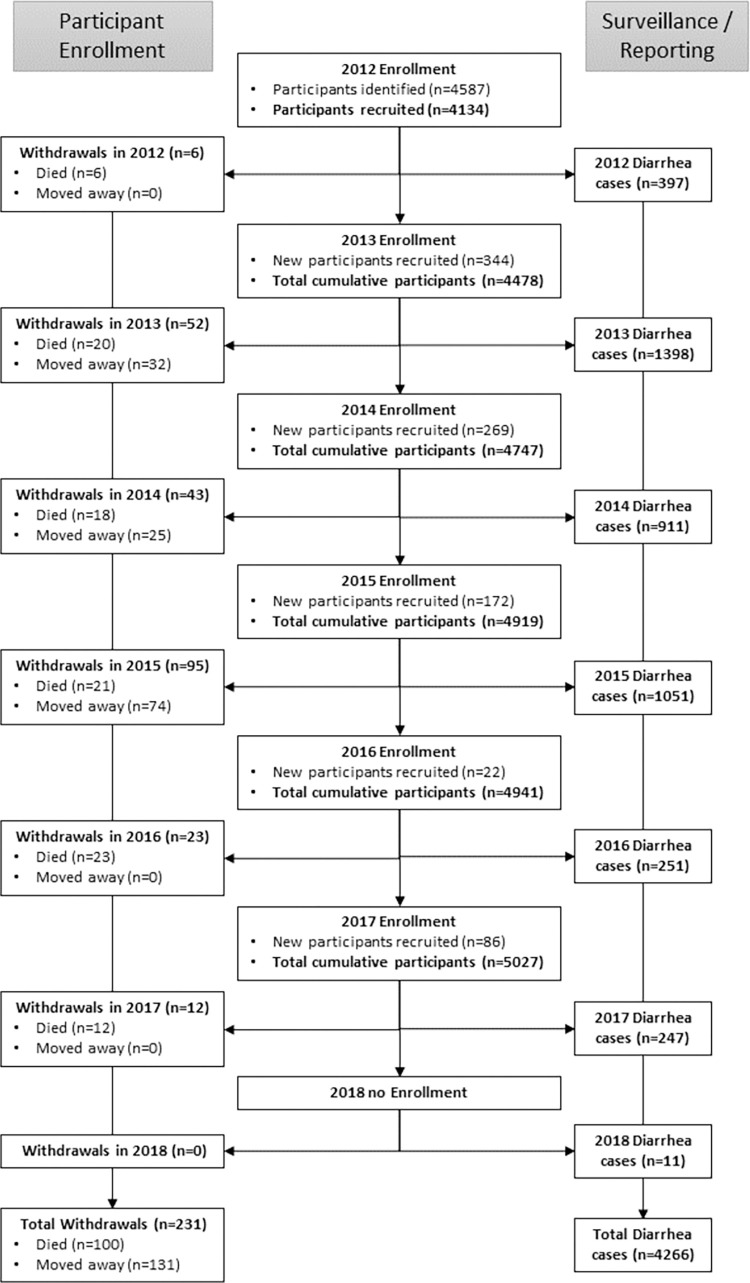
Flow chart illustrating participant enrolment and surveillance reporting workflows throughout the study period.

A total of 5027 participants were included in the study, from a potential 5500 individuals surveyed across 1139 households. Among those enrolled, 455 (8.27%) individuals did not meet the inclusion criteria and 18 (0.33%) refused to participate and were excluded from the study. Throughout the course of the five and one-half-year study period, 231 (4.60%) participants were not followed through the conclusion of the study either due to death (100; 1.99%) or moving away from their respective study village (131; 2.61%). A total of 4266 diarrhea cases were reported during the study. Fig 2 provides a flow-chart illustration of participation, rolling recruitment, and diarrhea cases captured throughout the study period.

Table 1 provides a breakdown of participant demographics. Of the 5027 total study participants, 2490 (49.5%) reported a FVBI, acute respiratory, or diarrheal disease illness as part of the acute surveillance program and were classified as a patient. A total of 1450 (28.8%) reported at least one diarrhea case during the study. Over the course of the study period, the diarrhea incidence rate was calculated at 281.5 persons per 1000 population per year, with an event rate of 664.3 individual diarrhea events occurring per 1000 population per year.

**Table 1:** Participant demographics.

	Category	Total Participants (n = 5027)	95% CI	p value
**Gender**	Female	2642 (52.6%)	[51, 54]	**<0.001**
** **	Male	2385 (47.4%)	[46, 49]	** **
**Age**	Mean (SD]	25.7 (20.1)	[25, 26]	
**Age Group**	Adult (≥18 yrs.]	2901 (57.5%)	[56, 59]	**<0.001**
** **	Children (<18 yrs.]	2126 (42.3%)	[41, 44]	** **
**Age Breakdown**	<5 yrs.	734 (14.6%)	[14, 16]	**<0.001**
	5–15 yrs.	1113 (22.1%)	[17, 19]	
	15–25 yrs.	902 (17.9%)	[15, 17]	
	25–35 yrs.	808 (16.1%)	[9.5, 11]	
	35–45 yrs.	519 (10.3%)	[7.4, 8.9]	
	45–55 yrs.	408 (8.1%)	[21, 23]	
** **	>55 yrs.	543 (10.8%)	[10, 12]	** **
**Population**	Peri-urban	3466 (68.9%)	[68, 70]	**0.011**
** **	Rural	1561 (31.1%)	[30, 32]	** **
**Water Source**	Protected	2336 (46.5%)	[45, 48]	**0.001**
** **	Unprotected	2691 (53.5%)	[52, 55]	** **
**Water Treated**	Treated	1,188 (23.6%)	[22, 25]	**<0.001**
	Untreated	1,302 (25.9%)	[25, 27]	
	No Data	2,537 (50.5%)	[49, 52]	
**Sanitation**	No facility	3796 (75.5%)	[74, 77]	**0.002**
** **	With facility	1231 (24.5%)	[23, 26]	** **
**Animal Expose**	Yes	2430 (48.3%)	[47, 50]	**<0.001**
	No	60 (1.2%)	[0.92, 1.5]	
	No Data	2537 (50.5%)	[49, 52]	
**Season visit**	Dry	462 (9.2%)	[8.4, 10]	**<0.001**
	Rainy	964 (19.2%)	[18, 20]	
	Both	1064 (21.2%)	[20, 22]	
	No Data	2537 (50.5%)	[49, 52]	

Among this cohort, 65.5% (1,094; [95% CI 63%, 68%]; p<0.001) of participants living in peri-urban settings reported at least one case of diarrhea during the study period, as compared to 43.4% (356; [95% CI 40%, 47%]; p<0.001) living in rural settings. Diarrhea was reported by 71.8% (934; [95% CI 69%, 74%], p<0.001) of participants without access to a protected water source. By age breakdown, only the 5–15 years age group had less than 50% of participants with an acute illness (305; 41.2% [95% CI 38%, 45%], p<0.001) reporting at least one case of diarrhea. Table 2 provides a summary of study participants with an acute illness that reported at least one case of diarrhea.

**Table 2:** Description of study patients reporting at least one case of diarrhea.

		Diarrhea Case Reported			
	Category	No n (%) [95%CI]	Yes n (%] (95%CI]		P value
**Gender**	Female	546 (40.5%) [38, 43]	801 (59.5%) [57%, 62%]		0.213
** **	Male	494 (43.2%) [40%, 46%]	649 (56.8%) [54%, 60%]		
**Age**	Mean (SD)	19.8 (18.2) [19, 21]	25.1 (21.5) [24, 26]		**<0.001**
**Age Group**	Adult (≥18 yrs.)	388 (33.4%) [31%, 36%]	774 (66.6%) [64%, 69%]		**<0.001**
** **	Children (<18 yrs.)	652 (49.1%) [46%, 52%]	676 (50.9%) [48%, 54%]		** **
**Age**	<5 yrs.	150 (31.9%) [28%, 36%]	320 (68.1%) [64%, 72%]		**<0.001**
**Breakdown**	5–15 yrs.	435 (58.8%) [55%, 62%]	305 (41.2%) [38%, 45%]		
	15–25 yrs.	163 (49.5%) [44%, 55%]	166 (50.5%) [45%, 56%]		
	25–35 yrs.	92 (31.4%) [26%, 37%]	201 (68.6%) [63%, 74%]		
	35–45 yrs.	72 (32.6%) [27%, 39%]	149 (67.4%) [61%, 73%]		
	45–55 yrs.	48 (26.7%) [20%, 34%]	132 (73.3%) [66%, 80%]		
** **	>55 yrs.	80 (31.1%) [26%, 37%]	177 (68.9%) [63%, 74%]		** **
**Population**	Peri-urban	576 (34.5%) [32%, 37%]	1,094 (65.5%) [63%, 68%]		**<0.001**
** **	Rural	464 (56.6%) [53%, 60%]	356 (43.4%) [40%, 47%]		** **
**Water**	Protected	673 (56.6%) [54%, 59%]	516 (43.4%) [41%, 46%]		**<0.001**
**Source**	Unprotected	367 (28.2%) [26%, 31%]	934 (71.8%) [69%, 74%]		** **
**Water**	Treated	375 (43.0%) [40%, 46%]	498 (57.0%) [54%, 60%]		0.387
**Treated**	Untreated	665 (41.1%) [39%, 44%]	952 (58.9%) [56%, 61%]		
**Sanitation**	No facility	395 (46.6%) [43%, 50%]	452 (53.4%) [50%, 57%]		0.318
** **	With facility	645 (39.3%) [37%, 42%]	998 (60.7%) [58%, 63%]		
**Animal**	No	54 (40.6%) [32%, 49%]	79 (59.4%) [51%, 68%]		0.821
**Expose**	Yes	986 (41.8%) [40%, 44%]	1,371 (58.2%) [56%, 60%]		
**Season visit**	Dry	325 (31.1%) [28%, 34%]	720 (68.9%) [66%, 72%]		**<0.001**
	Rainy	715 (49.5%) [47%, 52%]	730 (50.5%) [48%, 53%]		
**Total**		1040 (41.8%)	1450 (58.2%)		** **

Study participants with diarrhea had an average of 2.9 individual diarrhea events during the study period. The average length of time between each diarrhea event for a study patient was 1.4 years (p<0.001). Seasonality did not appear to influence the occurrence of multiple diarrhea case events, with 54.8% of study participants reporting diarrhea events in both the dry and wet seasons as opposed to only a specific season (p<0.001).

There were 4266 individual diarrhea case events reported in the study. Table 3 provides a summary of calculated incidence rates of individual diarrhea case events by category per 1000 population per year in the study. Incidence rates by gender were higher among females (295.81; [95% CI 275.67, 317.02], p = 0.05) than males. By age group, the highest incidence rates were recorded in the 35–45 years old category (433.81 [95% CI 362.96, 514.44], p<0.001) followed by adults over 55 years old (427.69; [95% CI 367.00, 495.55], p<0.001). Incidence rates were higher among individuals classified as unskilled workers (313.63; [95% CI 290.51, 338.10], p<0.001) or unemployed (282.78; [95% CI 261.88, 304.91], p<0.001); in peri-urban settings 347.35; [95% CI 327.07, 368.56], p<0.001); and among individuals using unprotected water sources (413.01; [95% CI 386.94, 440.37], p<0.001). Higher incidence rates were also recorded in the dry season compared to the wet season (392.86; [95% CI 364.69, 422.64], p<0.001).

**Table 3:** Diarrhea case incidence rates by category (per 1000 per year).

	Category	Incidence rate [95% CI]	p
**Gender**	Female	295.81 [275.67, 317.02]	**0.05**
** **	Male	265.58 [245.53, 286.82]	** **
**Age Group**	<5 yrs.	291.93 [260.82, 325.73]	**<0.001**
	5–15 yrs.	235.00 [200.61, 273.59]	
	15–25 yrs.	344.96 [298.91, 396.08]	
	25–35 yrs.	354.02 [299.46, 415.65]	
	35–45 yrs.	433.81 [362.96, 514.44]	
	45–55 yrs.	187.42 [166.98, 209.68]	
** **	>55 yrs.	427.69 [367.00, 495.55]	** **
**Occupation**	Skilled worker	156.95 [126.52, 192.48]	**<0.001**
	Unskilled worker	313.63 [290.51, 338.1]	
** **	Unemployed	282.78 [261.88, 304.91]	** **
**Population**	Peri-urban	347.35 [327.07, 368.56]	**<0.001**
** **	Rural	177.82 [159.82, 197.28]	** **
**Water Source**	Protected	178.54 [163.46, 194.63]	**<0.001**
** **	Unprotected	413.01 [386.94, 440.37]	
**Water Treated**	Treated	305.59 [279.34, 333.65]	**<0.001**
** **	Untreated	270.30 [253.40, 288.03]	** **
**Sanitation**	No facility	323.34 [303.59, 344.04]	**<0.001**
** **	With facility	218.88 [199.16, 240.02]	** **
**Animal Expose**	No	430.64 [340.94, 536.70]	**<0.001**
	Yes	275.96 [261.54, 290.96]	** **
**Season**	Dry	392.86 [364.69, 422.64]	**<0.001**
	Wet	219.95 [204.28, 236.50]	** **

### Frequency of single and mixed infections

Of the 4266 diarrhea cases recorded, 3841 cases, consisting of 3839 individual rectal swabs and 3764 individual fresh stools, were collected for analysis. Fresh stool samples or rectal swabs could not be collected during in-home medical assessments or within 48-hours in 425 cases due to patients being unable to provide or return a sample within the requisite time-period. From the 3841 diarrhea cases analyzed, 2726 (71.0%) were identified as positive for at least one stool pathogen (virus, parasite, or bacteria). Positivity rates were also statistically higher among females (72.8%; p = 0.002), children (76.1%; p<0.001), and patients without access to sanitation facilities (72.7%; p = 0.004). Mixed infections, defined as infections where two or more pathogens were detected, occurred in 36.2% of all positive cases. The most common type of mixed infection was parasite and bacterial, accounting for 69.8% of all mixed infections. Statistically higher rates of mixed infections were found in peri-urban areas (36.9%; p = 0.037), among children (41.5%; p<0.001), and during the dry season (40.8%; p<0.001). Table 4 provides a summary of stool specimen sample characteristics.

**Table 4:** Summary of stool specimen sample characteristics.

Pathogen identification	Total	Study site		Season		Gender		Age group		Drinking water source		Drinking water treatment		Sanitation	
		Peri-urban	Rural	Dry	Wet	Female	Male	Children	Adult	Un- protected	Protected	Un-treated	Treated	No facility	With facility
Total stool sample tested	3,841	3,306	535	1,798	2,043	2,354	1,487	1,559	2,282	2,350	1,491	1,823	2,018	2,326	1,515
Not Detected	1,115	950	165	421	694	640	475	372	743	698	417	556	559	636	479
Positive	2,726	2,356	370	1,377	1,349	1,714	1,012	1,187	1,539	1,652	1,074	1,267	1,459	1,690	1,036
*%Positive*	*71*.*0%*	*71*.*3%*	*69*.*2%*	*76*.*6%*	*66*.*0%*	*72*.*8%*	*68*.*1%*	*76*.*1%*	*67*.*4%*	*70*.*3%*	*72*.*0%*	*69*.*5%*	*72*.*3%*	*72*.*7%*	*68*.*4%*
*Sig*. *and OR [95%CI]*	* *	*p = 0*.*320*, *1*.*1 [0*.*9–1*.*4]*		*p<0*.*001*, *1*.*7 [1*.*5–1*.*9]*		*p = 0*.*002*, *1*.*3 [1*.*1–1*.*5]*		*p<0*.*001*, *1*.*5 [1*.*3–1*.*8]*		*p = 0*.*249 0*.*9 [0*.*8–1*.*1]*		*p = 0*.*060*, *0*.*9 [0*.*8–1*.*0]*		*p = 0*.*004*, *1*.*2 [1*.*1–1*.*4]*	
Pathogen identified:															
Single infection (parasite/bacteria/virus)	1,740	1,486	254	815	925	1,082	658	694	1,046	1,045	695	808	932	1,079	661
Mixed infection	986	870	116	562	424	632	354	493	493	607	379	459	527	611	375
*%Mixed infection*	*36*.*2%*	*36*.*9%*	*31*.*4%*	*40*.*8%*	*31*.*4%*	*36*.*9%*	*35*.*0%*	*41*.*5%*	*32*.*0%*	*36*.*7%*	*35*.*3%*	*36*.*2%*	*36*.*1%*	*36*.*2%*	*36*.*2%*
*Sig*.	* *	*p = 0*.*037*		*p<0*.*001*		*p = 0*.*320*		*p<0*.*001*		*p = 0*.*440*		*p = 0*.*954*		*p = 0*.*982*	
*Stool parasites*, *bacteria*, *and viruses*	*98*	*84*	*14*	60	38	60	38	40	58	69	29	50	48	71	27
*Stool parasites and bacteria*	*688*	*616*	*72*	376	312	450	238	379	309	415	273	326	362	420	268
*Stool parasites and viruses*	*109*	*99*	*10*	64	45	72	37	46	63	63	46	46	63	65	44
*Stool bacteria and viruses*	*91*	*71*	*20*	62	29	50	41	28	63	60	31	37	54	55	36

### Bacterial gastrointestinal infections

Of the 3839 stool samples tested, 1460 (38.0%) samples were positive for enteric bacteria. Positivity percentages were statistically higher among patients living in rural settings (42.5%; p = 0.022), females (39.5%; p = 0.016), and children (42.4%; p<0.001). Positivity percentages collected during the dry season (43.3%; p<0.001) were significantly higher than those collected during the wet season (33.4%). 374 of 1460 (25.6%) of positive samples were found to have more than one GI bacteria species in their stool samples (Table 5). Overall, pathogenic *Escherichia coli* (559; 38.3%), *Aeromonas spp*. (367; 25.1%), *Plesiomonas shigelloides* (353; 24.2%), and *Campylobacter spp*. (256, 17.5%) were the predominant bacteria. Through further molecular typing of 99% of the *E*. *coli* collected (558 out of 559), the predominant pathotypes were identified as enteroaggregative *E*. *coli* (EAEC) (206; 36.9%), enteropathogenic *E*. *coli* (EPEC) (193; 34.6%), and enterotoxigenic *E*. *coli* (ETEC) (149; 26.7%).

**Table 5:** Pathogens identified by laboratory testing type and stool specimen sample characteristics.

Pathogen identification	Total	Study site		Season		Gender		Age group		Drinking water source		Drinking water treatment		Sanitation	
		Peri-urban	Rural	Dry	Wet	Female	Male	Children	Adult	Un- protected	Protected	Un-treated	Treated	No facility	With facility
**Stool ova and parasite testing**						** **	** **								
Stool sample tested	3,764	3,257	507	1,780	1,984	2,315	1,449	1,516	2,248	2,305	1,459	1,779	1,985	2,268	1,496
Positive	1,936	1,732	204	950	986	1,232	704	823	1,113	1,180	756	894	1,042	1,206	730
%Positive	51.4%	53.2%	40.2%	53.4%	49.7%	53.2%	48.6%	54.3%	49.5%	51.2%	51.8%	50.3%	52.5%	53.2%	48.8%
*P-value and OR [95%CI]*	* *	*p<0*.*001*, *1*.*7 [1*.*4–2*.*1]*		*p = 0*.*025*, *1*.*2 [1*.*1–1*.*3]*		*p = 0*.*006*, *1*.*2 [1*.*1–1*.*4]*		*p = 0*.*004*, *1*.*2 [1*.*1–1*.*4]*		*p = 0*.*709 1*.*0 [0*.*9–1*.*1]*		*p = 0*.*170*, *0*.*9 [0*.*8–1*.*0]*		*p = 0*.*009*, *1*.*2 [1*.*1–1*.*4]*	
Detected with single species	1,266	1,122	144	628	638	782	484	522	744	764	502	587	679	782	484
Detected with two or more species	670	610	60	322	348	450	220	301	369	416	254	307	363	424	246
**Stool bacteria testing**															
Stool sample tested	3,839	3,305	534	1,798	2,041	2,352	1,487	1,559	2,280	2,349	1,490	1,821	2,018	2,325	1,514
Positive	1,460	1,233	227	779	681	930	530	661	799	887	573	696	764	911	549
%Positive	38.0%	37.3%	42.5%	43.3%	33.4%	39.5%	35.6%	42.4%	35.0%	37.8%	38.5%	38.2%	37.9%	39.2%	36.3%
*P-value and OR [95%CI]*	* *	*p = 0*.*022*, *0*.*8 [0*.*7–0*.*9]*		*p<0*.*001*, *1*.*5 [1*.*3–1*.*7]*		*p = 0*.*016*, *1*.*2 [1*.*1–1*.*4]*		*p<0*.*001*, *1*.*4 [1*.*2–1*.*6]*		*p = 0*.*665 1*.*0 [0*.*9–1*.*1]*		*p = 0*.*818*, *1*.*0 [0*.*9–1*.*2]*		*p = 0*.*070*, *1*.*1 [1*.*0–1*.*3]*	
Detected with one species	1,086	912	174	545	541	694	392	481	605	636	450	491	595	664	422
Detected with two or more species	374	321	53	234	140	236	138	180	194	251	123	205	169	247	127
Stool virus testing*															
Stool sample tested	3,720	3,242	478	1,735	1,985	2,280	1,440	1,500	2,220	2,271	1,449	1,750	1,970	2,226	1,494
Positive	414	345	69	270	144	244	170	254	160	261	153	186	228	255	159
%Positive	11.1%	10.6%	14.4%	15.6%	7.3%	10.7%	11.8%	16.9%	7.2%	11.5%	10.6%	10.6%	11.6%	11.5%	10.6%
*P-value and OR [95%CI]*	* *	*p = 0*.*014*, *0*.*7 [0*.*5–0*.*9]*		*p<0*.*001*, *2*.*4 [1*.*9–2*.*9]*		*p = 0*.*298*, *0*.*9 [0*.*7–1*.*1]*		*p<0*.*001*, *2*.*6 [2*.*1–3*.*2]*		*p = 0*.*378 1*.*1 [0*.*9–1*.*4]*		*p = 0*.*361*, *0*.*9 [0*.*7–1*.*1]*		*p = 0*.*441*, *1*.*1 [0*.*9–1*.*3]*	
Detected with one species	402	336	66	260	142	240	162	242	160	253	149	184	218	250	152
Detected with two or more species	12	9	3	10	2	4	8	12	0	8	4	2	10	5	7

* Tested only for Norovirus and Rotavirus (subject’s age ≤6 years old)

### Parasitic gastrointestinal infections

Of the 3764 stool samples tested for ova and parasites, 1936 (51.4%) were positive. Positivity percentages were statistically higher among participants living in peri-urban settings (53.2%; p<0.001), females (53.2%; p = 0.006), children (54.3%; p = 0.004), and patients without access to sanitation (53.2%; p = 0.009). Positivity percentages of samples collected during the dry season were also statistically significant (53.4%; p = 0.025) when compared to those collected during the wet season (49.7%). 670 of 1936 (34.6%) positive samples had more than one gastrointestinal (GI) parasite species detected in their stool sample (Table 5). Twenty-six (26) different gastrointestinal parasite species (13 helminths and 13 protozoa) were detected in the stool samples. Hookworm (133; 7%), *Strongyloides stercoralis* (133; 7%), and *Clonorchis/Opisthorchis* (120; 6.2%) were the predominant helminths species, while *Blastocystis hominis* (1039; 53.7%) and *Giardia lamblia* (425; 22.0%) were the predominant protozoan species.

### Viral gastrointestinal infections

A total of 414 (11.1%) of 3720 laboratory tested samples were positive for Norovirus and/or Rotavirus. Positivity percentages were statistically higher among patients living in rural settings (14.4%; p = 0.014) and children (16.9%; p<0.001). Positivity percentages of samples collected during the dry season were also significant (15.6%; p<0.001) compared to samples collected during the wet season (7.3%). 12 of 414 (2.9%) samples were positive with both Norovirus and Rotavirus. Norovirus GII was the predominant virus identified. Additionally, ten Norovirus positive individuals displayed no symptoms at the time of illness.

A summary of all pathogens identified in the study, their detection frequency and association with mixed infections is provided as supporting information (S1 Table).

### Risk analysis for gastrointestinal illness

Table 6 provides a summary risk analysis of the overall likelihood of acquiring diarrhea in our study population. Results from the study indicated males were 12% less likely to have risk of diarrhea compared to females (HR = 0.88 [95% CI 0.83, 0.94], p<0.001). In comparison with skilled workers, being unemployed increased the risk of contracting diarrhea by 63% (HR = 1.63 [95% CI 1.46, 1.83], p<0.001). Individuals without access to protected water sources (HR = 1.59 [95% CI 1.49, 1.69], p<0.001) or sanitation facilities (HR = 1.19 [95% CI 1.12, 1.28], p<0.001) were 59% and 19% at greater risk of contracting diarrhea, respectively. The wet season reduced the diarrhea hazard risk by 32% (HR = 0.68 [95% CI 0.64, 0.72], p<0.001).

**Table 6:** Cox survival regression model of likelihood of acquiring diarrhea.

Variable	Beta [95% CI]	HR [95% CI]	SE	z	p
**Gender**					
Female [ref]	–	–	–	–	–
Male	-0.13 [-0.19, -0.07]	0.88 [0.83, 0.94]	0.03	-3.99	**<0.001**
**Age**	0.01 [0.01, 0.01]	1.01 [1.01, 1.01]	<0.01	17.86	**<0.001**
**Occupation**					
Skilled worker [ref]	–	–	–	–	–
Unskilled worker	0.15 [0.03, 0.27]	1.16 [1.03, 1.31]	0.06	2.51	0.01
Unemployed	0.49 [0.38, 0.61]	1.63 [1.46, 1.83]	0.06	8.32	**<0.001**
**Population**					
Peri-urban [ref]	–	–	–	–	–
Rural	-0.83 [-0.91, -0.75]	0.44 [0.40, 0.47]	0.04	-19.97	**<0.001**
**Water Source**					
Protected [ref]	–	–	–	–	–
Unprotected	0.46 [0.40, 0.53]	1.59 [1.49, 1.69]	0.03	13.84	**<0.001**
**Water Treat**					
Treated [ref]	–	–	–	–	–
Untreated	0.09 [0.03, 0.16]	1.10 [1.03, 1.17]	0.03	2.73	0.006
**Sanitation**					
With facility [ref]	–	–	–	–	–
No facility	0.18 [0.11, 0.24]	1.19 [1.12, 1.28]	0.03	5.32	**<0.001**
**Animal Expose**					
No [ref]	–	–	–	–	–
Yes	-0.27 [-0.42, -0.11]	0.76 [0.65, 0.89]	0.08	-3.39	**<0.001**
**Season**					
Dry [ref]	–	–	–	–	–
Wet	-0.39 [-0.45, -0.33]	0.68 [0.64, 0.72]	0.03	-12.41	**<0.001**

**Fig 3:**
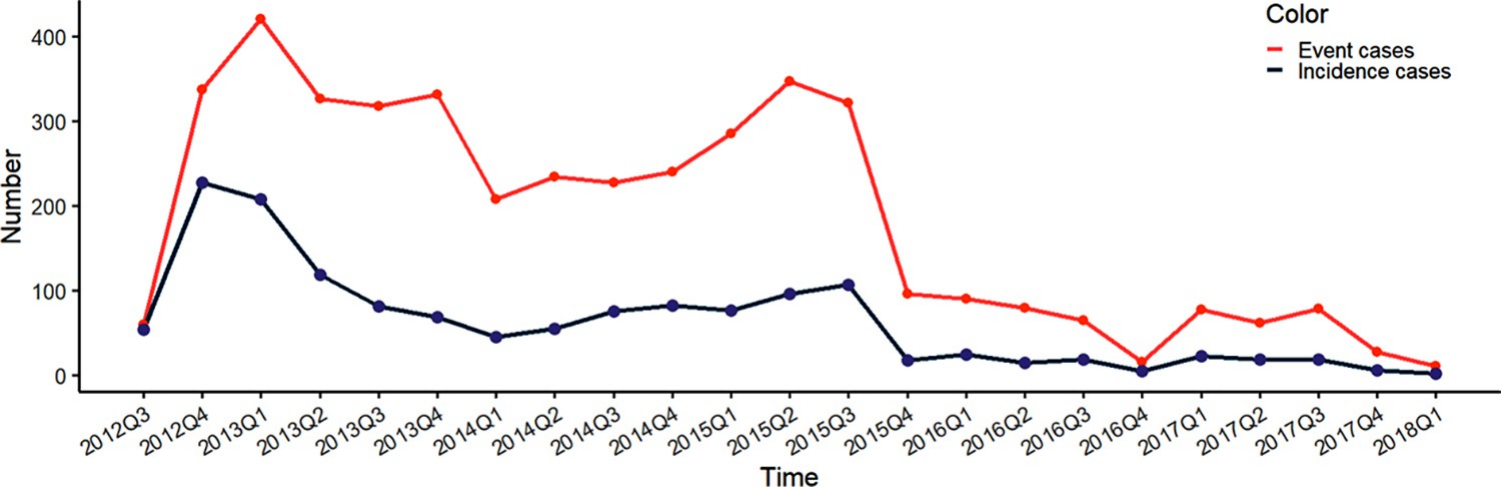
Diarrhea incidence (person per thousand population) and event (cases per thousand population) rates over the course of the study (Q3 2012 to Q1 2018).

Two distinct peaks in incidence and event rates during the study (Fig 3), with the first peak towards the end of 2012 and the second occurring in the third quarter of 2015. Following the second peak in 2015, both incidence and event rates declined sharply and remained at lower levels for the remainder of the study period relative to rates recorded from 2012 to 2015. *Blastocystis hominis* (96 cases), *Entamoeba histolytica/dispar* (58), *Pathogenic Escherichia coli* (49), *Shigella sp*. (48), *Aeromonas sp*. (43), *Plesiomonas shigelloides* (40), and *Norovirus* (39) were the predominant pathogens identified during the first peak of 2013/2014, while *Microsporidia spp*. (37), *Blastocystis hominis* (31), *Giardia lamblia* (28), *Campylobacter spp*. (15), and pathogenic *Escherichia coli* (11) were the dominant pathogens detected during the second peak in 2015.

## Discussion

The results from this study present an evaluation of diarrheal disease as part of a five and one-half-year active surveillance study among a targeted population living in both peri-urban and rural settings in Cambodia. Key methodologic aspects of this long-term study, including the collection of detailed individual participant and household-level information, the adoption of both passive and active case-finding surveillance approaches, and the incorporation of laboratory-level diagnostics, allowed for the detection of diarrheal pathogens and detailed epidemiological data which could have been missed through typical passive case detection measures only. Similarly, the longitudinal component of this study allowed for the assessment of temporal changes of disease incidence and enabled patient-level tracking and recording of multiple infection events, providing insight into the trends and burden of diarrheal disease within the community.

With almost one in three study participants experiencing diarrhea, an event rate of 664.3 individual diarrhea events per 1000 population per year, and an average of 2.9 diarrhea case events among individuals reporting diarrhea, the impact of these diseases on the population are clear. Data presented in Table 2 indicate that diarrheal infections impacted both adults and children alike, with more than half of patients in each age category (except the 5–15-year age group) recording at least one diarrhea case in the study. Given the burden of diarrheal diseases, including the potential impacts these illnesses have on growth and development during early childhood, and in terms of DALYs [2, 23–27], understanding the likelihood of exposure events within a population has important considerations for health program planning.

In line with previous research [28–30], results from this study identified a statistical association between a participant reporting diarrhea and the use of unprotected water sources. Several related risk factors consistent with previous studies are also highlighted, including poor water quality and being unemployed [31–33], as well as findings linked to socioeconomic factors including living conditions, access to amenities and services, and education. Living in a peri-urban setting was also statistically associated with patients reporting diarrhea compared to patients living in a rural environment in this study. As standards among urban living conditions vary considerably based on a multitude of factors such as socioeconomics, levels of governance, and culture, specific data regarding peri-urban vs rural settings and diarrhea are somewhat limited. However, previous research on living conditions, crowding, and sanitation have demonstrated an increased association of diarrhea [34–36], and may be factors to consider for targeted public health interventions. Of note, while no statistical association between diarrhea and access to sanitation facilities was found in the bivariate analyses in this study, this factor had a calculated diarrhea hazard risk of 19% as part of the multi-event survival regression modelling. Previous research into sanitation and diarrhea indicated that additional factors such as maintaining cleanliness of sanitation facilities and promoting handwashing and good hygiene practice may be required to support a reduction in diarrhea, beyond simply improving access [31, 35, 36], suggesting further detailed investigation into these aspects of sanitation would be beneficial for health programs.

Seasonality also impacted the risk of acquiring diarrhea. Despite a higher number of actual diarrhea cases reported during wet season periods over the course of the study period, the calculated incidence rate of dry season infections was higher. Similarly, results of the multi-variate analysis identified a reduction in the hazard risk of acquiring diarrhea during the wet season by 32%. Potential reasoning associated with these findings may include factors such as the concentration-dilution hypothesis where rainfall events occurring after dry periods may flush pathogens into surface water, whereas rainfall following wet periods may have a dilutive impact of pathogen concentrations [37]. In populations where large proportions of individuals lack access to protected water sources, such as in this study, these findings provide valuable insight for identifying at risk populations and potential high-risk transmission periods. Further research is required to analyze the potential causative factors contributing to increased prevalence of diarrhea.

Several species of pathogenic *E*. *coli* (ETEC, EPEC, and EAEC) are highly infectious to humans, and since such a high percentage of acute infections in our study were found to contain these bacterial species, it is reasonable to deduce that they were likely the primary causative agent even among the many mixed infections, including those with co-infection or colonization with other pathogens such as *Blastocystis hominis*. Species of pathogenic *E*. *coli* in this study were found to be more common in children during the dry season in peri-urban areas closer to animals and areas of food production, which is logical as pathogenic *E*. *coli* is a food-borne bacteria known to cause serious GI illness. Poorly cooked or raw foodstuffs can contain ETEC, EPEC, and/or EAEC, along with other bacteria and/or parasites; therefore, better food safety initiatives in peri-urban areas could lead to fewer incidents of GI illness, especially in children. *Shigella* infections resulted in 43 (26%) of all bloody diarrhea samples collected in this study, and therefore is important to understand when diagnosing GI illness and considering antibiotic treatment and a patient presents with hematemesis or bloody dysentery. The water-borne parasite *Giardia spp*., which is known to be found in contaminated food, soil, and water tainted with animal feces was, unsurprisingly, found most frequently in children in our study. Parasitic disease prevention programs, including comprehensive education for young people regarding personal hygiene and properly cooking food and sterilizing water, is critical to reduce acute GI illness in non-urban areas.

As illustrated in Fig 3, a key finding from the study was the observed decline in overall diarrheal incidence over the course of the study period, with a significant reduction recorded between the third and fourth quarter of 2015. This reduction and the subsequent low incidence rate reported through to the end of the study period could potentially suggest a beneficial impact resulting from a targeted intervention; however, further investigation would be required to validate this. Of note, early during this surveillance study, in 2014, was the commencement of yearly meetings between the study site Provincial Health Departments and the Cambodia Ministry of Health to review data generated from the study and discuss diarrhea prevention programs. An example targeted response interventions derived from these yearly meetings included the deployment of health personnel in 2016 to Kampong Cham province to investigate diarrheal infection and subsequently recommend interventions including the targeted chlorination of water sources, and implementation of healthy food preparation and safe storage education and awareness programs [38]. Further research is required to confirm the impacts of such targeted prevention and control interventions on diarrhea incidence within Cambodia as this study was not designed to determine the effectiveness of an intervention, but instead to observe and describe incidence.

Limitations associated with this study include potential bias associated with the self-reporting component of the study methodology. While efforts were made by study personnel to adequately guide household primary contacts on illness identification and reporting procures, and to confirm self-reported illnesses during the routine weekly visits, the potential for the misidentification of potential cases should be noted. Given the length of the study, from a statistical perspective several factors were likely to have changed over time that could not be accounted for in the regression modelling. The regression model analysis used the last state of these factors, and as such does not indicate an association in the change of factors to the outcomes over time.

Aligning the etiologies of diarrheal disease with an understanding of the epidemiological and demographic risk profiles and transmission characteristics within a population provided valuable public health insight. Given the global significance of diarrheal disease, particularly in low and middle-income countries where health systems and disease surveillance systems are often strained, studies that support the identification and characterization of etiologies and associated risk factors leading to infection are essential. Data generated from this study provides evidence-based data for health programs to strengthen the planning and the delivery of appropriate and targeted community and population-specific interventions accordingly.

## Supporting information

S1 Questionnaire Inclusivity in global research questionnaire. (DOCX)

S1 Table Summary of pathogens detected, their frequency, and association with mixed infections. (DOCX)
