# Supplementary material for: Etiology and risk factors for diarrheal disease amongst rural and peri-urban populations in Cambodia, 2012–2018
Source: PLoS One. 2023 Mar 31;18(3):e0283871. doi: 10.1371/journal.pone.0283871 (PMC10065300; doi:10.1371/journal.pone.0283871)
Supplement: S1 Table — (DOCX) [file pone.0283871.s002.docx]

**S1 Table.** **Summary of pathogens detected, their frequency, and association with mixed infections.**

| **Stool pathogen** | **Frequency detection (n)** | **Single infection** | **Coinfection (Dual/Triple/more)** |  |
| --- | --- | --- | --- | --- |
|  |  |  |  |  |
| **Parasitic GI pathogen** | | | |  |
| **Helminths** |  |  |  |  |
| *Ascaris lumbricoides* | 1 | 0 | 1 |  |
| *Ascaris spp.* | 17 | 7 | 10 |  |
| *Capillaria phillipinensis* | 1 | 0 | 1 |  |
| *Clonorchis sinensis* | 44 | 16 | 28 |  |
| *Clonorchis/Opisthorchis* | 120 | 54 | 66 |  |
| *Dicrocoelium spp.* | 2 | 2 | 0 |  |
| *Enterobius vermicularis* | 5 | 1 | 4 |  |
| *Fasciolopsis buski* | 18 | 5 | 13 |  |
| Hook worm | 133 | 58 | 75 |  |
| *Hymenolepis diminuta* | 1 | 1 | 0 |  |
| *Hymenolepis nana* | 35 | 9 | 26 |  |
| *Strongyloides stercoralis* | 133 | 39 | 94 |  |
| *Trichuris trichiura* | 4 | 3 | 1 |  |
| **Protozoa** |  |  |  |  |
| *Blastocystis hominis* | 1039 | 521 | 518 |  |
| *Cryptosporidium spp.* | 72 | 30 | 42 |  |
| *Cyclospora cayetanensis* | 1 | 0 | 1 |  |
| *Dientamoeba fragilis* | 1 | 0 | 1 |  |
| *Endolimax nana* | 3 | 1 | 2 |  |
| *Entamoeba coli* | 282 | 80 | 202 |  |
| *Entamoeba histolytica* | 2 | 0 | 2 |  |
| *Entamoeba histolytica/dispar* | 268 | 77 | 191 |  |
| *Giardia lamblia* | 425 | 245 | 180 |  |
| *Iodamoeba buetschii* | 2 | 0 | 2 |  |
| *Microsporidia spp.* | 197 | 116 | 81 |  |
| *Sarcocystis spp* | 15 | 1 | 14 |  |
| *Trichomonas hominis* | 2 | 0 | 2 |  |
| **Bacterial GI pathogen** | | | |  |
| *Aeromonas spp.* | 367 | 170 | 197 |  |
| *Arcobacter cryaerophilus* | 1 | 1 | 0 |  |
| *Campylobacter spp.* | 287 | 178 | 109 |  |
| *Pathogenic Escherichia coli* | 559 | 375 | 184 |  |
| *Plesiomonas shigelloides* | 353 | 198 | 155 |  |
| *Salmonella spp.* | 11 | 6 | 5 |  |
| *Shigella sp.* | 267 | 140 | 127 |  |
| *Vibrio alginolyticus* | 4 | 2 | 2 |  |
| *Vibrio cholerae non O1 non O139* | 17 | 8 | 9 |  |
| *Vibrio cholerae Ogawa* | 1 | 0 | 1 |  |
| *Vibrio fluvialis* | 1 | 1 | 0 |  |
| *Vibrio parahaemolyticus* | 20 | 11 | 9 |  |
| *Yersinia pestis* | 2 | 0 | 2 |  |
| **Viral GI pathogen** | | | |  |
| Norovirus | 365 | 353 | 12 |  |
| Rotavirus | 61 | 49 | 12 |  |
